# Role of hepatoma‐derived growth factor in promoting *de novo* lipogenesis and tumorigenesis in hepatocellular carcinoma

**DOI:** 10.1002/1878-0261.12357

**Published:** 2018-08-07

**Authors:** Xuejie Min, Jun Wen, Li Zhao, Kaiying Wang, Qingli Li, Gang Huang, Jianjun Liu, Xiaoping Zhao

**Affiliations:** ^1^ Department of Nuclear Medicine Ren Ji Hospital School of Medicine Shanghai Jiao Tong University China; ^2^ Shanghai University of Medicine & Health Sciences China

**Keywords:** HCC, HDGF, lipid metabolism, SREBP

## Abstract

Although identified as a growth factor, the mechanism by which hepatoma‐derived growth factor (HDGF) promotes cancer development remains unclear. We found that nuclear but not cytoplasmic HDGF is closely associated with prognosis of hepatocellular carcinoma (HCC). RNA‐sequencing analysis further demonstrated that the nuclear role of HDGF involved regulation of transcription of lipid metabolism genes. HDGF‐induced expression of lipogenic genes was mainly associated with activation of sterol regulatory element binding protein (SREBP) transcription factor. Coexpression of SREBP‐1 and nuclear HDGF predicts poor prognosis for HCC. In addition, by changing the first amino acid of the PWWP domain from proline to alanine, the type of PWWP domain changed from P‐ to A‐type, resulting in inability to induce SREBP‐1‐mediated gene transcription. The type of PWWP domain affects the recruitment of the C‐terminal binding protein‐1 transcriptional repressor on the promoter of the lipogenic gene. Our data indicate that HDGF acts as a coactivator of SREBP1‐mediated transcription of lipogenic genes. The PWWP domain is crucial for HDGF to promote lipogenesis. Moreover, transcriptional regulation of nuclear HDGF plays important roles in the development of HCC.

**Abbreviations**
Acac: acetyl‐CoA carboxylase
Fasn: fatty acid synthase
HCC: hepatocellular carcinoma
HDGF: hepatoma‐derived growth factor
Scd1: stearoyl‐CoA desaturase 1
SREBP: sterol regulatory element binding protein

## 1 Introduction

Liver cancer is the third leading cause of cancer death worldwide. The global incidence of liver cancer, especially in Asia, continues to rise, with China accounting for > 50% of the global burden (Petrick *et al*., 2016; Uhlen *et al*., 2017). The most common histological type of primary liver cancer is hepatocellular carcinoma (HCC) (Akinyemiju *et al*., 2017). Studying the molecular mechanisms that lead to the development and progression of HCC is crucial for identifying new targets for early diagnosis and treatment.

To maintain uncontrolled growth, cancer cells undergo complex metabolic reprogramming, involving energy generation and macromolecular biosynthesis. Metabolic disorders have been identified as a hallmark of cancer (Hanahan and Weinberg, 2011). Activation of *de novo* lipogenesis is found in many types of malignancy, including HCC (Bhalla *et al*., 2012; Jacobs *et al*., 2012; Liu *et al*., 2016; Rysman *et al*., 2010). In cancer cells, unrestricted lipogenesis is essential for the continued supply of lipids and lipid precursors to maintain membrane production, synthesis of signal transducers, and post‐translational modification of proteins (Menendez and Lupu, 2007; Swinnen *et al*., 2006). Since hepatocytes are mainly responsible for lipid synthesis and storage, lipid metabolism plays a more important role in the development of liver cancer. *De novo* lipogenesis is gradually induced from nontumor liver tissue to liver cancer. Blocking lipogenesis is a potential strategy for targeted therapy of HCC (Calvisi *et al*., 2011). Genetic ablation of lipogenic enzymes results in complete inhibition of HCC development in mice with an AKT overexpression background (Li *et al*., 2016a). Targeted treatment options for HCC are limited. Therefore, there is an urgent need to understand the underlying mechanism of uncontrolled lipogenesis in HCC.

At the molecular level, synergistic increased expression of fatty acid synthase (*Fasn*), stearoyl‐CoA desaturase 1 (*Scd1*), acetyl‐CoA carboxylase (*Acac*), and other lipogenic enzymes results in increased *de novo* lipogenesis. Overexpression of lipogenic enzymes is closely related with hepatocarcinogenesis (Hu *et al*., 2016; Li *et al*., 2016a). These lipogenic enzymes are primarily transcriptionally activated by sterol regulatory element binding protein (SREBP) (Goldstein *et al*., 2006). There are three SREBP proteins, SREBP‐1a, 1c, and 2 in mammals. SREBP‐1a and c are produced by the same gene *Srebf1* through two different promoters, while *Srebf2* encodes SREBP‐2 (Sato, 2010). SREBP‐1 mainly activates genes involved in fatty acid and triglyceride synthesis, whereas SREBP‐2 is involved in cholesterol synthesis (Jeon and Osborne, 2012). As the predominant form in cancer cells, SREBP‐1a is a potent activator of all SREBP‐responsive genes, including those that mediate fatty acid, triglyceride, and cholesterol synthesis (Horton *et al*., 2002). Increased lipid synthesis is a hallmark of tumor cells and depends primarily on SREBP‐mediated transcription (Menendez and Lupu, 2007; Shao and Espenshade, 2012; Shimano and Sato, 2017). Compared with adjacent normal tissues, SREBP‐1 expression is high in HCC tissues. The positive expression of SREBP‐1 is correlated with poor survival of HCC patients (Li *et al*., 2014). SREBP transcriptional activity is generally low in normal and nonproliferating cells, which typically import lipids from the extracellular environment. In contrast, actively proliferating cells, particularly tumor cells, have an increased demand for lipids, which largely depends on *de novo* lipogenesis. Sequencing of HCC tissue and nontumor tissue indicates that the target of SREBP‐1 is generally activated in cancer tissues (Calvisi *et al*., 2011). Therefore, SREBP transcriptional activity is crucial for tumor growth, making it a potential therapeutic target (Shao and Espenshade, 2012). It is important to understand how cancer cells activate SREBP‐mediated gene transcription, which may translate into therapeutic strategies by effectively targeting lipid metabolism.

hepatoma‐derived growth factor (HDGF) is a heparin‐binding acidic glycoprotein that was originally identified from conditioned serum‐free medium by a human hepatoma‐derived cell line HuH‐7 and exhibits mitogenic activity in various cell types (Ren *et al*., 2009). HDGF is a member of a family of growth factors called HDGF‐related proteins, consisting of a highly conserved N‐terminal 100‐residue PWWP domain, also called the HATH domain (homologous to the N terminus of HDGF) and a disordered C‐terminal 140‐residue domain (Chen *et al*., 2015a). Recent evidence indicates that the first residue of the PWWP motif in HDGF regulates the PWWP domain structure, protein stability, and protein–protein interactions (Hung *et al*., 2015). HDGF is a broad regulator of cancer cell activity and is involved in many cellular processes including transformation, apoptosis, angiogenesis, and metastasis (Bao *et al*., 2014b). Although HDGF has been identified as an oncogene, the mechanism by which HDGF promotes tumor development is not fully understood. It is noteworthy that HDGF is able to translocate to the nucleus where it binds to DNA through its PWWP domain as a transcription cofactor (Chen *et al*., 2015b). However, little is known about the role of HDGF in the nucleus.

Here, we investigated the role of HDGF in lipid metabolism of HCC. Our results show that HDGF overexpression is an indicator of poor prognosis of HCC. HDGF expression leads to upregulation of lipogenic enzyme expression and subsequent lipid biosynthesis. The type of PWWP domain is important for HDGF to regulate lipid metabolism. Mutating PWWP domain from A‐ to P‐type caused HDGF to fail to promote lipogenesis in HCC cells. Mechanistically, HDGF acts as a coactivator of SREBP‐1‐mediated transcription of lipogenic genes. In addition, coexpression of HDGF and SREBP‐1 is positively correlated with poor prognosis in HCC patients. Our data suggest that HDGF and SREBP‐1 synergistically promote HCC development by activating lipid biosynthesis. The HDGF/SREBP‐1 axis might be used to develop new diagnostic and therapeutic targets.

## 2 Materials and methods

### 2.1 Cell culture

The cells were purchased from Cell Bank of the Chinese Academy of Science (Shanghai, China). HepG2, HEK293T, and 7721 were cultured in Dulbecco's modified Eagle's medium (DMEM; Gibco, Carlsbad, CA, USA) supplemented with 10% FBS (Gibco), 100 mg·mL^−1^ penicillin (Gibco), and 100 mg·mL^−1^ streptomycin (Gibco), under 5% CO_2_ in a humidified incubator at 37 °C. For low‐lipid culture conditions, charcoal‐stripped FBS (Gibco) was used instead of FBS.

### 2.2 Transfection of siRNA

Cells were transfected with siRNAs using Lipofectamine 2000 (Invitrogen, Waltham, MA, USA). The sequences of siRNA were as follows: HDGF (sense 5ʹ‐CGAGAACAACCCUACUGUCAA‐3ʹ, antisense 5ʹ‐ UUGACAGUAGGGUUGUUCUCG‐3ʹ), CtBP1 (sense 5ʹ‐CCACGCCAGTGACCAGTTGTA‐3ʹ, antisense 5ʹ‐TACAACTGGTCACTGGCGTGG‐3ʹ), and negative control (sense 5ʹ‐UUCUCCGAGCGUGUCACGUTT‐3ʹ, antisense 5ʹ‐ACGUGACACGUUCGGAGAATT‐3ʹ).

### 2.3 Quantitative real‐time PCR (qPCR)

Total RNA was extracted using the TRIzol kit (Omega, Norcross, GA, USA), and cDNA was synthesized using the Prime‐Script RT kit (Takara, Dalian, China). After reverse transcription, the cDNA was amplified on a QuantStudio™ real‐time PCR system (Applied Biosystems, Waltham, MA, USA) using SYST Green PCR Master Mix (Takara, Shiga, Japan). The PCR program was predenatured at 95 °C for 10 s, annealed at 60 °C for 30 s, and extended for 40 s for 40 cycles before melting curve analysis. Relative mRNA levels were calculated using the 2^−ΔΔCt^ method. Each experiment was performed in triplicate, including a negative control. The primers are listed in Table 1.

**Table 1:** Primers used for qPCR

Gene	Forward primer (5′–3′)	Reverse primer (5′–3′)
HDGF	CTCTTCCCTTACGAGGAATCCA	CCTTGACAGTAGGGTTGTTCTC
FASN	AGTACACACCCAAGGCCAAG	GGATACTTTCCCGTCGCATA
SREBF‐1	GCTGCTGACCGACATCGAA	CCAGCATAGGGTGGGTCAAA
SREBF‐2	AGGCAGGCTTTGAAGACGAA	GTACATCGGAACAGGCGGAT
ACLY	CAGTCCCAAGTCCAAGATCCC	GTCTCGGGAGCAGACATAGT
ACSS2	TCGGCCTGTTTTCTCAGTCC	GTCTCCCCAGAATTCCCGC
ELOVL2	CGCTGCGGATCATGGAACAT	AGCATGTACGCGGAGAGAAG
ELOVL5	CGCTTGATTCATCCTTCGGG	CTAGTATCTCGAGGGCCTAGCA
ELOVL6	GCTAAGCAAAGCACCCGAAC	GGAGCACAGTGATGTGGTGA
SCD	CACTTGGGAGCCCTGTATGG	TGAGCTCCTGCTGTTATGCC
SCD5	CCCTGGTACATCTGGGGAGA	GAAGCCTTCACCAATGGCAC
FADS1	CAAATCCACTCCTGGAGCCC	CACAAAGGGATCCGTGGCAT
LDLR	GGTCCACATTTGCCACAACC	ATGTTCACGCCACGTCATCC
ACAT2	AACTGCTAGGTGGTCTGAGC	CACCATTGAAGGAACACCTGC
HMGCR	GCCCTCAGTTCCAACTCACA	TTCAAGCTGACGTACCCCTG
HMGCS1	TGTCCTTTCGTGGCTCACTC	GGCATGGTGAAAGAGCTGTG
HMGCS2	CTGGGATGGTCGTTATGCCA	TATTGGGTACTCCGAGGCCA
MVK	CCAGGAGCCATGTTGTCAGA	TACAGCCAGTGCTACCTTGC
MVD	TCAAGTACTGGGGCAAGCG	CAAATCCGGTCCTCGGTGAA
SQLE	TTTCTGGGCATTGCCACTTTC	ATTGGTTCCTTTTCTGCGCCTC
AACS	AAGAACACGCAGATGGACCG	TCATAACTCTCCAGCGCCAG
DHCR7	GAGGTGTGCGCAGGACTTTA	CCCTTGAGATGCGGTTCTGT
FDPS	AGAGCGGGAACTACTCGACC	GAGCAAAGGGCTCGAGGTTC
HSD17B7	TTGGCCATTTTATCCTGATTCGG	GCTGTAGGGTTCCTTGCCTT
NSDHL	CGCCTACGGACGGAAAAGA	CGTGCGACTTGGTCTCTCAT
PMVK	CTCCCCATATCTGTTTGGACA	CAGCTCCAAGTCTGCTCTG
LSS	CGGAGGGCACGTGTCTG	GCAGCCCCACGTAAAATGTC
ACACA	CATCTCCACCCCTGTTGCAG	TCCAAAAAGACCTAGCCCTCAA
GPAM	GAAGCTGGAGCTGCTAGGG	CCACACTCACCCCATTCCTC
CYP51A1	CTCGTTCCGTCGATTGGGAG	TGTATGGAGGACTTTTCACCCC
FDFT1	GACTCGACAGACTCTAAGGCTC	TGGTCAATAAGTCGCCCACG
IDH1	CAGGCTGTGGTTGTGAGTCT	TAGTTTATCGCCTGCCGGG
STARD4	ACGTCCTTGCTTCACCTCAG	ACACCTTGGGCTTTGTAGAGAT
TM7SF2	TGCCTCATCAATGCTACTGGTTA	CACTCTGGGGTCAGAAGGATT
ALDOC	CCGCAGCCTCATTTACCAGA	CATGGTGACAGCTCCCTGTG
FADS2	CACGGGGCGTCACAGTC	AAGGCATCCGTTGCATCTTCT
TMEM97	AGTCGAGTTTAGAAACCTGCTGA	GCTGAAACACAAGCTCGCAA
ALDH1A2	TAGGGACACCCGGCCC	TATCTGCCTTGTCTGCTTCTTGA
CB1	AGATGTAGGCCGGGTGATCT	CCGCCCTGGATCATGAAGTC

### 2.4 Western blotting

Cells were washed with cold PBS and lysed in RIPA buffer [1 mm dl‐dithiothreitol, 0.25 mm PMSF, 2 μg·mL^−1^ aprotinin, and 1 mm 4‐(2‐aminoethyl)‐benzenesulfonyl fluoride] with protease inhibitors for 30 min on ice, followed by centrifugation at 15 000 ***g*** for 30 min. Protein concentration was determined with the bicinchoninic acid assay. Samples were loaded on 10% SDS/PAGE, separated by electrophoresis, and transferred to polyvinylidene difluoride membranes. Membranes were blocked with 5% milk in Tris‐buffered saline Tween‐20 for 1 h at room temperature and then incubated with antibodies overnight at 4 °C. The membranes were then incubated with secondary antibodies for 1 h at room temperature. Antibodies used for western blotting included anti‐HDGF (1 : 1000; Proteintech, Rosemont, IL, USA), anti‐CtBP1 (1 : 1000; Proteintech), anti‐β‐tubulin (1 : 2000; Cell Signaling Technology, Boston, MA, USA), anti‐β‐actin (1 : 2000; Proteintech), anti‐GAPDH (1 : 2000; Proteintech), anti‐glutathione *S*‐transferase (GST; 1 : 1000; Abcam, Cambridge, MA, USA), anti‐6 ×  His (1 : 1000; Proteintech), anti‐Flag M2 (1 : 2000; Sigma‐Aldrich, Burlington, MA, USA). Goat anti‐mouse (1 : 15 000; LI‐COR, Lincoln, NE, USA) and goat anti‐rabbit (1 : 15 000; LI‐COR) were used as secondary antibodies. The signal was detected with the Odyssey infrared imaging system.

### 2.5 Coimmunoprecipitation

Cells were washed with cold PBS and lysed in IP buffer [150 mm NaCl, 50 mm Tris/HCl (pH 7.5), 5 mm EDTA, 0.5% NP‐40, and 1% Triton X‐100] with protease inhibitor (0.25 mm PMSF) for 30 min on ice, followed by centrifugation at 15 000 ***g*** for 30 min. Cell extracts were incubated with anti‐Flag M2 affinity gel (Sigma‐Aldrich) in IP buffer for 3 hat 4 °C. After washing, samples were subjected to western blotting using specific antibodies.

### 2.6 RNA‐sequencing (RNA‐Seq) analysis

RNA of HepG2 cells stably expressing wild‐type (WT) or P24A mutant HDGF was extracted for RNA‐Seq analysis. Data analysis was performed as described previously (Li *et al*., 2016b). RNA degradation and contamination were monitored on 1% agarose gels. RNA purity was checked using the NanoPhotometer^®^ spectrophotometer (IMPLEN, Westlake Village, CA, USA). RNA concentration was measured using Qubit^®^ RNA Assay Kit in Qubit^®^ 2.0 Fluorometer (Life Technologies, Waltham, MA, USA). RNA integrity was assessed using the RNA Nano 6000 Assay Kit of the Bioanalyzer 2100 system (Agilent Technologies, Santa Clara, CA, USA). A total of 1.5 μg RNA per sample were used as input material for the RNA sample preparations. Sequencing libraries were generated using NEBNext^®^ Ultra™ RNA Library Prep Kit for Illumina^®^ (NEB, Ipswich, MA, USA). The clustering of the index‐coded samples was performed on a cBot Cluster Generation System using HiSeq 4000 PE Cluster Kit (Illumina, San Diego, CA, USA). After cluster generation, the library preparations were sequenced on an Illumina HiSeq 4000 platform and 150‐bp paired‐end reads were generated. Reference genome and gene model annotation files were downloaded from a genome website directly. Index of the reference genome was built using bowtie v2.2.9, and paired‐end clean reads were aligned to the reference genome using tophat v2.1.1. htseq v0.6.1 was used to count the read numbers mapped to each gene. The fragments per kilobase million value of each gene was calculated based on the length of the gene and read count mapped to this gene. Differential expression analysis of two conditions was performed using the desseq package (1.26.0). A *P* value of 0.05 was set as the threshold for significant differential expression.

### 2.7 Gene set enrichment analysis (GSEA)

Gene sets were from msigdb_v6.1 (Broad Institute) and manually curated from studies (Horton *et al*., 2003; Reed *et al*., 2008; Rome *et al*., 2008). Differentially expressed genes were rank‐ordered by fold change in expression. Gene set enrichment analysis (GSEA) was performed using default settings (GSEA preranked) (Subramanian *et al*., 2005).

### 2.8 Glutathione *S*‐transferase (GST) pull‐down assay

Glutathione *S*‐transferase‐tagged SREBP‐1a, GST alone, His‐HDGF, and HDGF derivatives were expressed in BL21‐DE3 cells (Sangon Biotech, Shanghai, China) under induction of 0.5 mmol·L^−1^ isopropyl β‐d‐1‐thiogalactopyranoside (Ameresco, Framingham, MA, USA) at 20 °C for 4 h. Cells were harvested by centrifugation at 3000 ***g*** for 10 min and resuspended in GST extraction buffer (20 mm HEPES, pH 7.6, 0.5 m NaCl, 0.5 μm EDTA, 10% glycerol, and 0.5% NP‐40). After sonicating for 20 minutes, the suspension was centrifuged at 12 000 ***g*** for 30 min at 4 °C. Glutathione–Sepharose 4B beads (GE Healthcare, Chicago, IL, USA) were added to the supernatant and incubated for 1 hat 4 °C. The beads were washed three times with GST Wash I (20 mm HEPES, pH 7.6, 1 m NaCl, 0.5 μm EDTA, 10% glycerol, and 1% NP‐40), five times with GST extraction buffer, and twice with GST Wash II (20 mm HEPES, pH 7.6, 100 mm KCl, 0.1 μm EDTA, 10% glycerol, and 0.02% NP‐40). All of these buffers were freshly added with 1 mm dl‐dithiothreitol, 0.25 mm PMSF, 2 μg·mL^−1^ aprotinin, and 1 mm 4‐(2‐aminoethyl)‐benzenesulfonyl fluoride. His‐tagged proteins were purified by Ni‐affinity resins (GE Healthcare). GST recombinant protein and His‐tagged proteins were incubated for 1 h at 4 °C. The resulting beads were washed three times with IP buffer and then subjected to western blot analysis with specific antibodies.

### 2.9 Soft agar colony formation assay

All cell lines (100 cells per well) were seeded in 24‐well plates. Cells were suspended in a mixture of 250 μL 0.8% agarose and DMEM (v : v = 1 : 1) and placed on the bottom agar composed of 0.6% low‐melt agarose in DMEM. The medium was changed regularly. After 14 days in culture, adherent cells were washed with PBS and fixed with 4% paraformaldehyde for 15 min at room temperature. Colonies were stained with 0.1% crystal violet for 30 min and then washed with PBS and air‐dried. Cell colonies were counted by imagej software (USA). Experiments were done in triplicate.

### 2.10 Cell viability assay

Cells were seeded in 96‐well plates and incubated until attached to the wells. Cells were cotransfected by siRNA against HDGF or control siRNA and HA‐tagged plasmid for 30 h. Cell viability was measured using Cell Counting Kit‐8 (Dojindo, Rockville, MD, USA) and normalized to untreated controls. The optical density was measured at 450 nm using a Bio‐Rad microplate 680 model (Hercules, CA, USA).

### 2.11 Luciferase reporter assay

The human *Fasn* promoter (1 kb) was cloned into pG13 vector (Promega, Madison, WI, USA). HepG2 cells were seeded in 24‐well plates at 1.5 × 10^4^ per well. Firefly luciferase plasmid and Renilla luciferase plasmid (as an internal control) were transfected into cells at a ratio of 10 : 1. Twenty‐four hours after transfection, cell lysates were analyzed by dual luciferase assay system (Promega). The ratio of firefly luciferase to Renilla activity was calculated for each of the triplicates.

### 2.12 Immunofluorescence staining

HepG2 cells stably expressing Flag‐tagged HDGF and HDGF P24A were transfected with HA‐tagged SREBP‐1a for 48 h. Cells were rinsed with PBS and fixed in 4% paraformaldehyde for 15 min at room temperature and washed three times with PBS. After permeabilization with 0.25% Triton X‐100 in PBS for 10 min at room temperature, the cells were blocked in 1% BSA in PBST (PBS and Tween‐20: 0.1%) for 30 min at room temperature. Cells were incubated with anti‐Flag antibody (1 : 500; Sigma‐Aldrich) and anti‐HA antibody (1 : 500; Sigma‐Aldrich) in PBST with 1% BSA for 1 h at room temperature in a humidified chamber. After washing, cells were incubated with goat anti‐mouse IgG (H + L) Alexa Fluor 568 (Invitrogen) and goat anti‐rabbit IgG (H + L) Alexa Fluor 488 (Invitrogen) at a dilution of 1 : 500 in 1% BSA in PBST for 1 h in the dark. The cell nuclei were visualized by staining with DAPI‐Fluoromount‐G (Southern Biotech, Birmingham, AL, USA). The slides were observed under a Zeiss Axioskop microscope (Oberkochen, Germany). The results were from two independent experiments, prepared in triplicate.

### 2.13 Oil red O staining

HepG2 cells were seeded in 24‐well plates. Prior to staining, cells were washed with PBS and fixed in 4% paraformaldehyde for 20 min at room temperature, washed three times with PBS, and permeabilized in 60% isopropanol for 10 s. Cells were stained for 30 min at room temperature in Oil Red O working solution and washed with 60% isopropanol for 10 s. The cells were washed three times with PBS and observed under a Zeiss Axioskop microscope.

### 2.14 Triglycerides and cholesterol assay

Cells transfected with siRNA‐HDGF (or siRNA‐NC as a control) for 48 h were washed three times with PBS and lysed in lysis buffer. Total protein concentration was determined using the bicinchoninic acid Protein Assay Kit (Abcam). Intracellular triglycerides and cholesterol were measured using the triglyceride assay kit (Biovision, Milpitas, CA, USA) and the cholesterol assay kit (Biovision) and normalized to the total protein concentration. Intracellular triglyceride and cholesterol levels were measured using a Bio‐Rad Model 680 microplate reader.

### 2.15 ChIP

ChIP analysis was performed using a commercial kit (Beyotime Biotechnology, China). Cells (4 × 10^7^) were fixed with 37% formaldehyde (final concentration 1%) at 37 °C for 10 min. Formaldehyde was quenched by adding glycine solution for 5 min at room temperature. Cells were harvested and lysed in SDS lysis buffer on ice for 10 min. The crude extract was sonicated to cut the chromosomal DNA to an average length of 200–1000 bp. Samples were precleared by incubation with Protein A/G agarose for 30 min at 4 °C. The primary antibody was anti‐HDGF (Proteintech), anti‐SREBF1 (Abcam), or IgG (Cell Signaling Technology) as a negative control. Protein A/G agarose was added and incubated overnight at 4 °C. Then, samples were washed with low salt immune complex wash buffer (once), high salt immune complex wash buffer (once), LiCL immune complex wash buffer (once), and TE buffer (twice). The DNA–protein complex was eluted in elution buffer, and 5 m NaCl (final concentration 200 mm) was added for reverse cross‐linking and incubation at 65 °C for 4 h. Purified DNA was used for quantitative (q)PCR assay. Primers were as follows: *Fasn* (sense 5ʹ‐CGACGCTCATTGGCCTGG‐3ʹ, antisense 5ʹ‐TGCCGTCTCTCTGGCTC‐3ʹ), *SCD* (sense 5ʹ‐TGGAAGAGAAGCTGAGAAGG‐3ʹ, antisense 5ʹ‐TTCTGTAAACTCCGGCTCGT‐3ʹ), *SYMD1* (sense 5ʹ‐TGCCTCAGCCTCCTCAGTAG‐3ʹ, antisense 5ʹ‐AAGCTAAACTGAGGGCTGGG‐3ʹ), and *GAPDH* (senseB 5ʹ‐TACTAGCGGTTTTACGGGCG‐3ʹ, antisense 5ʹ‐TCGAACAGGAGGAGCAGAGAGCGA‐3ʹ).

### 2.16 Xenograft study

All experimental procedures using animals were conducted in accordance with guidelines issued by the Animal Ethics Committee of Renji Hospital, Shanghai Jiaotong University School of Medicine (Shanghai, China). HepG2 cells stably expressing HDGF and P24A (5 × 10^6^ per injection) were injected subcutaneously into the shoulder sides of 15 male BALB/c nude mice (aged 4 weeks; Shanghai Laboratory Animal Center, Shanghai, China). The subcutaneous tumors were measured over 3 weeks. After killing, the tumors were weighed. The results are presented as the mean ± SEM.

### 2.17 Immunohistochemistry

All experiments involving human tissues were approved by the Human Assurance Committee of Renji Hospital, Shanghai Jiao Tong University School of Medicine. Immunostaining analysis was performed on resected paraffin‐embedded HCC tissues. Paraffin blocks with representative areas of the tumors were cut into 4‐μm‐thick sections that were processed for staining. Endogenous activity was quenched by incubation with 3% hydrogen peroxide for 30 min after deparaffinization and hydration. Antigen retrieval was subsequently carried out. The primary antibodies used were anti‐HDGF (1 : 200; Proteintech) and anti‐SREBP‐1 (1 : 1000; Santa Cruz Biotechnology, Dallas, TX, USA). Diaminobenzidine was used as a chromogen, and the slides were counterstained with hematoxylin. The slides were examined by two independent investigators who were blinded to the clinical characteristics of the patients. The percentage of positive staining was scored as 0 (0%–9%), 1 (10%–25%), 2 (26%–50%), 3 (51%–75%), or 4 (76%–100%) and the intensity as 0 (no staining), 1 (weak staining), 2 (moderate staining), or 3 (dark staining). The total score was calculated as the product of intensity and extent, ranging from 0 to 12.

### 2.18 Statistical analysis

Data were analyzed using graphpad Prism 6 software (GraphPad Software, USA) or spss 20.0 (SPSS, Dallas, TX, USA). Quantitative data are expressed as the mean ± SD of at least three independent experiments. Statistical differences between groups were assessed by the unpaired two‐tailed *t*‐test or analysis of variance. The chi‐square test was used for rate comparisons. Kaplan–Meier analysis was used in the survival duration assay. *P* < 0.05 was considered statistically significant.

## 3 Results

### 3.1 HDGF is an indicator of prognosis of HCC

**Figure 1:**
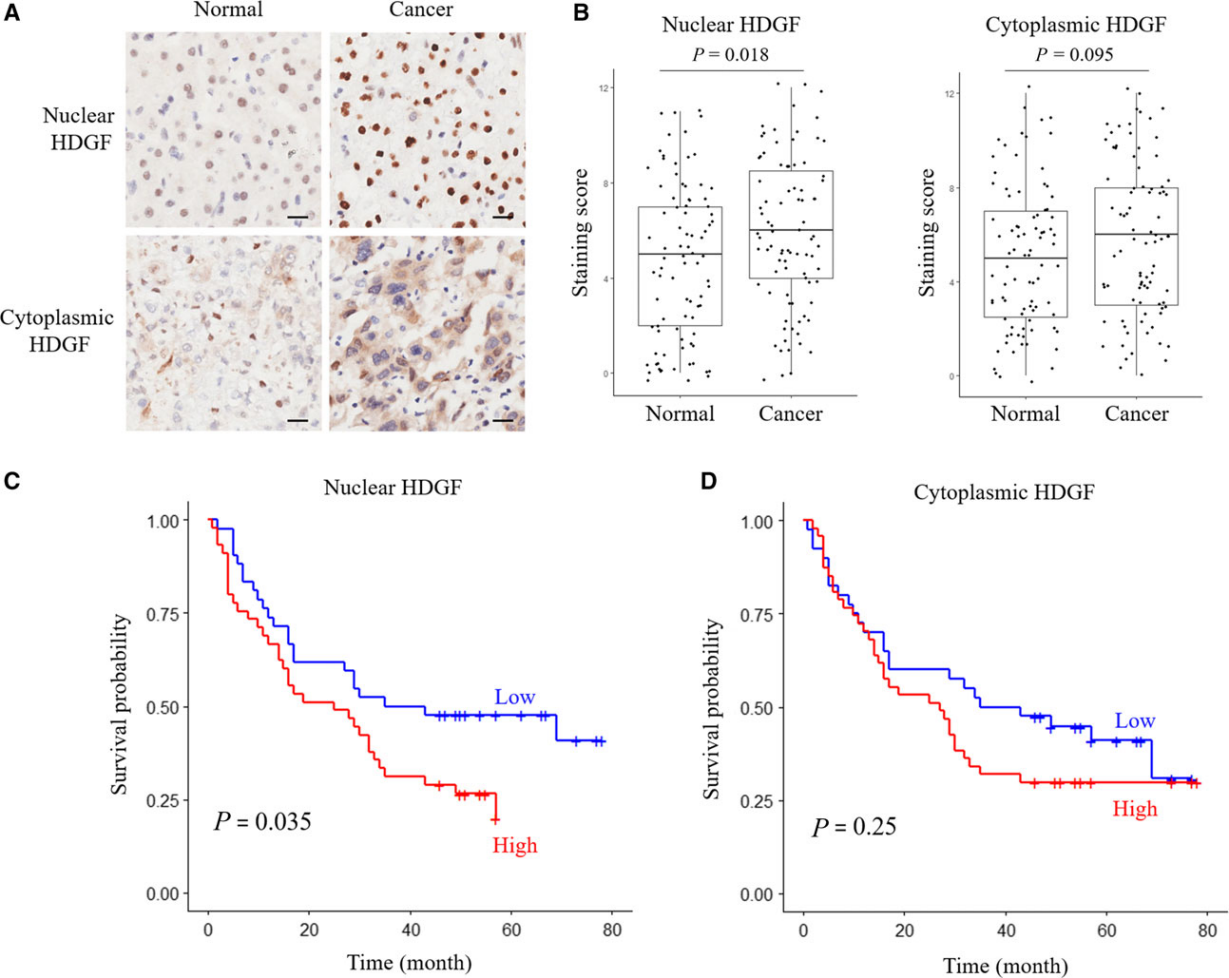
High expression of HDGF positively correlates with poor prognosis of HCC. (A) Representative images of a pair of HCC tissues (left) and adjacent normal tissues (right) stained with HDGF antibody (scale bar: 20 μm). (B) Statistical analysis of levels of nHDGF (left panel; staining score: 4.621 ± 3.361 and 5.862 ± 3.170 for normal and cancer, respectively) or cytoplasmic HDGF (right panel; staining score: 5.002 ± 3.087 and 5.839 ± 3.306 for normal and cancer, respectively) between HCC tissues and adjacent normal tissues. (C) Kaplan–Meier analysis of OS rates for HCC patients with high or low levels of nHDGF. (D) Kaplan–Meier analysis of OS rates for HCC patients with high or low levels of cytoplasmic HDGF.

Immunostaining analysis was performed to determine the expression profile of HDGF protein. HDGF expression was first examined in pairs of HCC and adjacent nontumor liver tissues. HDGF immunostaining was detected in the cytoplasm as well as in the nuclei of cells. Expression of HDGF was divided into low and high levels according to the median immunostaining score. Compared with adjacent nontumor liver tissues, there was a significant increase in nuclear HDGF (nHDGF) expression in cancer tissues (Fig. 1A,B). In contrast, HDGF cytoplasmic staining was higher, but not significantly, in HCC than adjacent normal tissues (Fig. 1A,B). We further analyzed the association of HDGF with clinicopathological parameters of HCC. Increased nuclear expression of HDGF was significantly correlated with poor differentiation and advanced clinical stage (Table 2). Increased cytoplasmic expression of HDGF was only associated with the advanced clinical stage of HCC (Table 2). Kaplan–Meier analysis showed that HCC patients with a high level of nuclear expression of HDGF had a significantly lower overall survival (OS) rate than those with low nuclear expression of HDGF (Fig. 1B). There was no significant correlation between cytoplasmic HDGF expression and HCC survival (Fig. 1C). These results suggest that increased nuclear expression of HDGF is closely associated with progression of HCC.

**Table 2:** Association of HDGF expression with characteristics of HCC

Characteristics	nHDGF expression			Cytoplasm HDGF expression		
	Low (%)	High (%)	*P*	Low (%)	High (%)	*P*
Gender						
Female	7 (8)	3 (3)	NS	5 (6)	5 (6)	NS
Male	35 (40)	42 (48)		35 (40)	42 (48)	
Age (year)						
< 60	30 (34)	34 (39)	NS	30 (34)	34 (39)	NS
≥ 60	12 (14)	11 (13)		10 (11)	13 (15)	
Tumor grade						
Good	6 (7)	2 (2)		4 (5)	4 (5)	
Moderate	30 (34)	26 (30	0.024	25 (29)	31 (36)	NS
Poor	6 (7)	17 (20)		11 (13)	12 (14)	
Maximal diameter (cm)						
< 5	22 (25)	15 (17)	NS	20 (23)	17 (20)	NS
≥ 5	20 (23)	30 (34)		20 (23)	30 (34)	
TNM stage						
I–II	26 (30	15 (17)	0.014	24 (28)	17 (20)	0.045
III–IV	16 (18)	30 (34)		16 (18)	30 (34)	

### 3.2 HDGF enriches for SREBP‐regulated lipogenic genes

**Figure 2:**
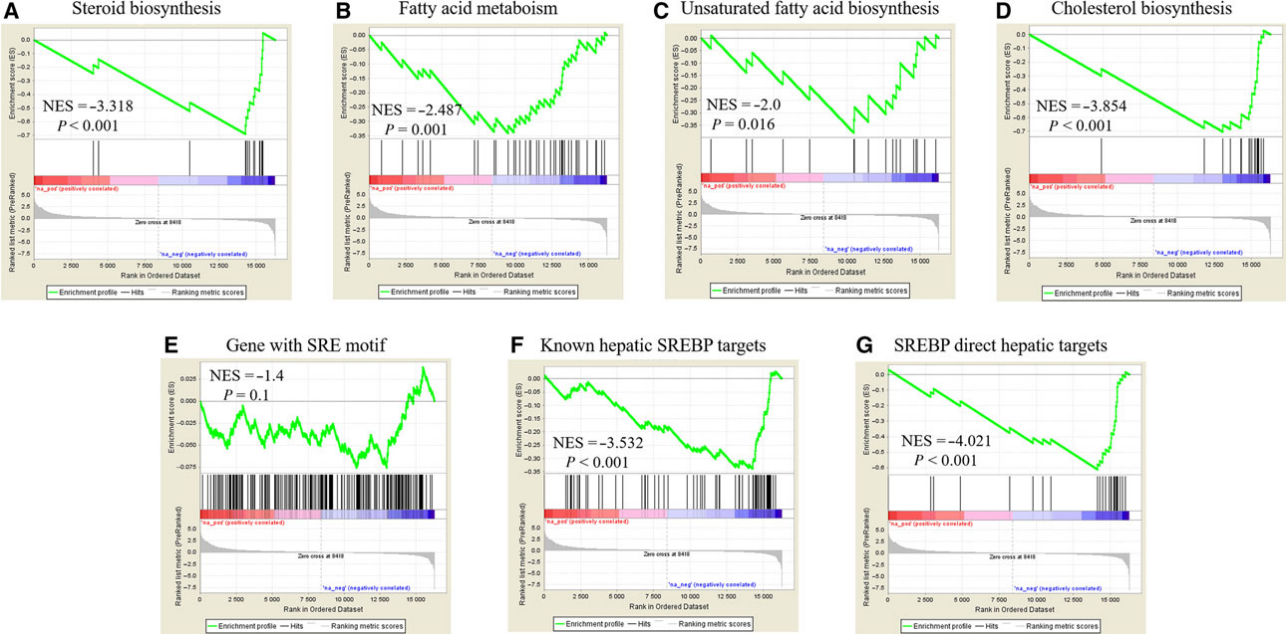
HDGF knockdown enriches genes involved in lipid metabolism. GSEA in HepG2 cells with HDGF knockdown. (A) Enrichment for KEGG_STEROID_BIOSYNTHESIS (NES = −3.318, *P* < 0.001). (B) Enrichment for KEGG_FATTY_ACID_METABOLISM (NES = −2.487, *P* = 0.001). (C) Enrichment for KEGG_BIOSYNTHESIS_OF_UNSATURATED_FATTY_ACIDS (NES = −2.0, *P* = 0.016). (D) Enrichment for REACTOME_CHOLESTEROL_BIOSYNTHESIS (NES = −3.854, *P* < 0.001). (E) Enrichment for genes with SRE motif (NES = −1.4, *P* = 0.1). (F) Enrichment for known hepatic SREBP targets (NES = −3.532, *P* < 0.001). (G) Enrichment for SREBP direct hepatic targets (NES = −4.021, *P* < 0.001).

To explore the functional role of HDGF in HCC, we analyzed RNA‐Seq data of HepG2 cells with HDGF knockdown derived from the ENCODE project (ENCODE Project Consortium, 2012). GSEA (Subramanian *et al*., 2005) was performed to identify biological features regulated by HDGF. HDGF knockdown showed significantly less enrichment of genes involved in lipid metabolism, including fatty acid metabolism, unsaturated fatty acid biosynthesis, steroid biosynthesis, and cholesterol biosynthesis pathways (Fig. 2A–D). SREBPs are key regulators of transcription of many enzymes required for lipid metabolism (Horton *et al*., 2002). We hypothesized that SREBPs may be involved in HDGF‐induced transcriptional regulation. To test this, we analyzed the enrichment of SREBP targets using GSEA. SREBP_Target_with_SRE gene signature (Rome *et al*., 2008), a set of genes with an SRE motif in the promoter, displayed a trend toward less enrichment in cells with HDGF knockdown (Fig. 2E). However, we found no significant association between HDGF and genes with the SRE motif, suggesting that not all SREBP targets were regulated by HDGF (Fig. 2E). The enrichment of transcripts associated with the Known_Hepatic_SREBP_Target gene signature (Reed *et al*., 2008) was significantly inhibited by HDGF knockdown (Fig. 2F). Furthermore, SREBP_Direct_Hepatic_Target gene signature (Horton *et al*., 2003), which is increased by *in vivo* overexpression of nuclear form SREBP‐1a (nSREBP‐1a) or nuclear form SREBP‐1 (nSREBP‐2), and decreased by *in vivo* SCAP knockout, was less enriched in cells with HDGF knockdown (Fig. 2G). Therefore, these data indicated that HDGF specially induced transcription of liver‐specific SREBP targets involved in fatty acid and cholesterol biosynthesis.

### 3.3 HDGF promotes SREBP‐1‐mediated gene transcription and lipid metabolism

**Figure 3:**
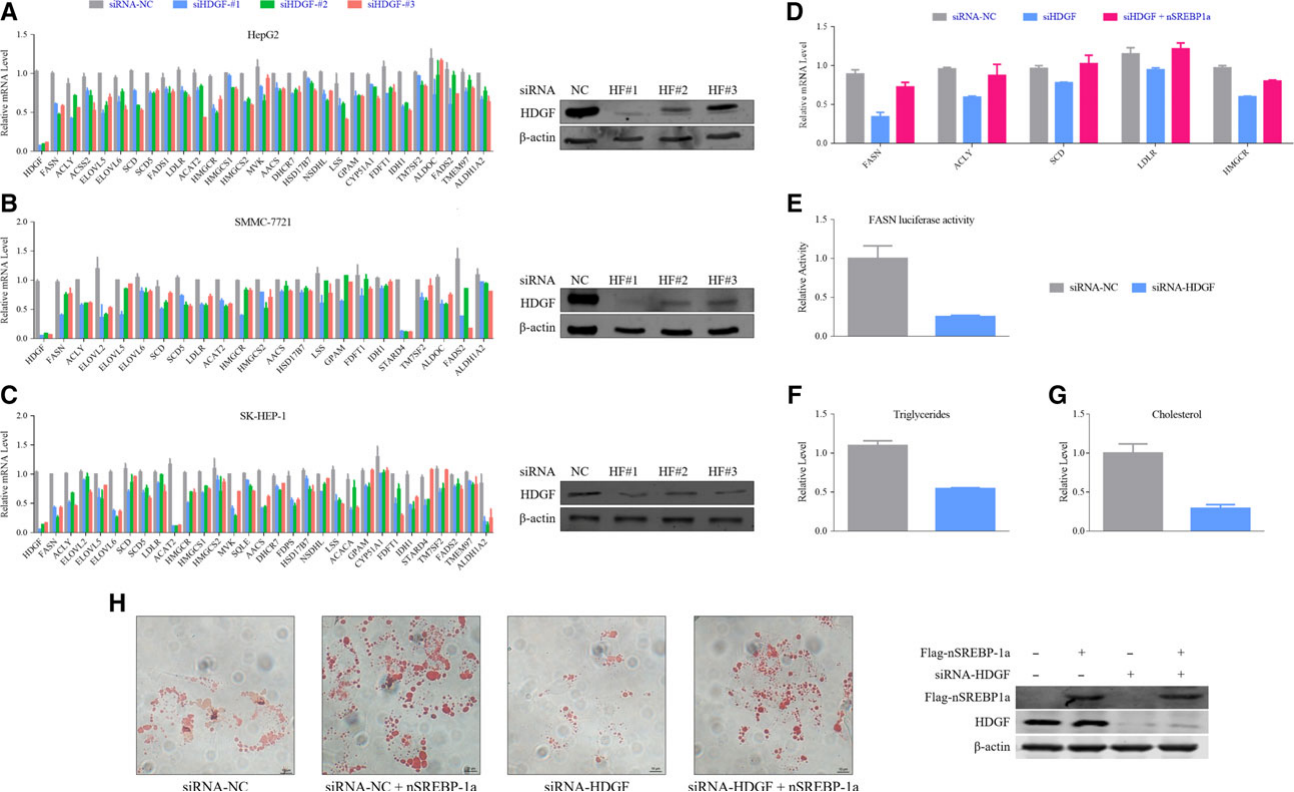
HDGF promotes SREBP‐1‐mediated gene transcription and lipid biosynthesis. (A) HepG2 cells were transfected with siRNA‐HDGF (or siRNA‐NC as control) for 48 h. mRNA levels of indicated genes were analyzed with qPCR. HDGF protein was analyzed with western blotting. β‐Actin was used as a loading control for western blotting. Error bars represent means ± SD (*n* = 3). (B) SMMC‐7721 cells were transfected with siRNA‐HDGF (or siRNA‐NC as control) for 48 h. mRNA levels of indicated genes were analyzed with qPCR. HDGF protein was analyzed with western blotting. β‐Actin was used as a loading control for western blotting. Error bars represent means ± SD (*n* = 3). (C) HepG2 cells were cotransfected with siRNA‐HDGF (or siRNA‐NC as control) and Flag‐SREBP‐1a (or empty vector as control) for 48 h. mRNA levels of indicated genes were analyzed with qPCR. Error bars represent means ± SD (*n* = 3). (D) HepG2 cells were cotransfected with siRNA‐HDGF (or siRNA‐NC as control) and FASN firefly luciferase reporter (Renilla luciferase reporter as internal control). The FASN reporter activity was calculated by firefly luciferase activity divided by Renilla luciferase activity. Error bars represent means ± SD (*n* = 3). (E) Cellular triglyceride levels were analyzed in HDGF stably knockdown HepG2 cells or control HepG2 cells. Triglyceride levels were normalized by total protein levels and the levels in control cells. Error bars represent means ± SD (*n* = 3). (F,G) Cellular triglycerides and cholesterol levels were analyzed in HDGF stably knockdown HepG2 cells or control HepG2 cells. Cholesterol levels were normalized by total protein levels and the levels in control cells. Error bars represent means ± SD (*n* = 3). (H) HepG2 cells were cotransfected with siRNA‐HDGF (or siRNA‐NC as control) and Flag‐SREBP‐1a (or empty vector as control) for 48 h. Right panel: Pictures of representative Oil Red O staining images were presented (Scale bar, 10 μm). Left panel: Protein levels of HDGF and Flag‐nSREBP‐1a were analyzed with western blotting. Error bars represent means ± SD (*n* = 3).

To validate the RNA‐Seq results, changes in mRNA expression of SREBP target genes in HCC cells were analyzed with qPCR. The knockdown efficiency of three siRNAs was determined with qPCR and immunoblotting (Fig. 3A–C). HDGF knockdown substantially decreased expression of most SREBP‐targeted genes, including *Fasn, Acly*,* Scd*, and others (Fig. 3A–C). All three HCC cell lines showed a similar phenotype. Some SREBP target genes were differently regulated by HDGF in different HCC cells. Overexpression of nSREBP‐1a rescued the inhibitory effect on SREBP target gene expression induced by HDGF knockdown (Fig. 3D). As expected, transient knockdown of *HDGF* in HepG2 cells decreased transcriptional activity of *Fasn* promoter (Fig. 3E). These results suggest that HDGF‐mediated expression of lipogenic genes is due to enhanced SREBP1 transcriptional activity. We investigated whether HDGF affected fatty acid and cholesterol biosynthesis in HCC cells. Compared with control cells, the levels of triglycerides and cholesterol in HDGF knockdown cells were significantly reduced (Fig. 3F,G). Lipid droplets comprising mainly triglycerides and sterol esters, as indicated by Oil Red O staining, were less abundant in cells with HDGF knockdown than in control cells (Fig. 3H). nSREBP‐1a overexpression reversed the decrease in lipids caused by HDGF knockdown (Fig. 3H), suggesting that SREBP‐1a is involved in HDGF‐regulated lipid biosynthesis. These results suggest that HDGF stimulates SREBP‐1‐mediated gene transcription and subsequent lipid metabolism.

### 3.4 P‐type PWWP domain of HDGF is critical for regulation of SREBP1‐mediated gene transcription

**Figure 4:**
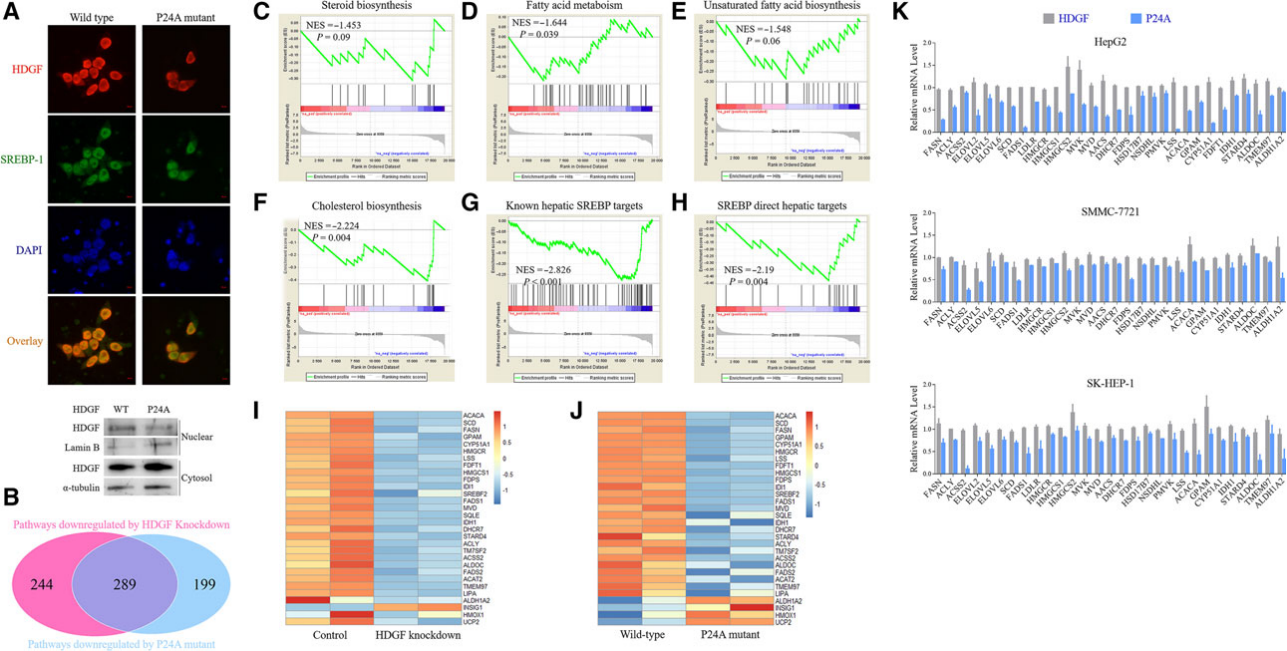
P24A mutation of HDGF decreases expression of lipogenic genes. (A) HepG2 cells were transfected with Flag‐HDGF (WT or P24A mutant and HA‐nSREBP‐1a for 48 h. Immunofluorescence was performed with antihemagglutinin and anti‐Flag antibodies. Upper panel: Coverslips were examined by confocal microscope (scale bar: 50 μm). Lower panel: Protein levels of HDGF in the nuclear or cytoplasmic fractions were analyzed with western blotting. (B) GSEA was performed for pathway analysis. Overlap pathway regulated by both HDGF knockdown and P24A mutation was displayed. GSEA in HepG2 cells with expressing WT or P24A mutant of HDGF. (C) Enrichment for KEGG_STEROID_BIOSYNTHESIS (NES = −1.453, *P* = 0.09). (D) Enrichment for KEGG_FATTY_ACID_METABOLISM (NES = −1.644, *P* = 0.039). (E) Enrichment for KEGG_BIOSYNTHESIS_OF_UNSATURATED_FATTY_ACIDS (NES = −1.548, *P* = 0.06). (F) Enrichment for REACTOME_CHOLESTEROL_BIOSYNTHESIS (NES = −2.224, *P* = 0.004). (G) Enrichment for known hepatic SREBP targets (NES = −2.826, *P* < 0.001). (H) Enrichment for SREBP direct hepatic targets (NES = −2.19, *P* = 0.004). (I) Heatmap displayed SREBP targets in HepG2 cells with HDGF knockdown. (J) Heatmap displayed SREBP targets in HepG2 cells with expression WT or P24A mutant of HDGF. (K) HepG2 cells stably expressing HDGF and P24A were cultured for 48 h. The mRNA levels of indicated genes were analyzed with qPCR. Error bars represent means ± SD (*n* = 3).

We next sought to explore the mechanisms underlying HDGF‐induced gene expression. HDGF is a unique growth factor and may act through either a receptor‐mediated pathway or a more direct way, that is, DNA, RNA, or protein binding(Chen *et al*., 2018; Hung *et al*., 2015; Rona *et al*., 2016). We found that HDGF mainly colocalized with SREBP‐1a within the nucleus, implying that nHDGF rather than receptor‐bound HDGF involved in SREBP‐mediated transcription (Fig. 4A). HDGF contains a conserved PWWP domain previously known as the HATH domain, which is required for binding with heparin, DNA, and protein (Chen *et al*., 2018; Hung *et al*., 2015; Rona *et al*., 2016). According to the first amino acid, the PWWP domain is classified into P‐type (Pro–His–Trp–Pro) and A‐type (Ala–His–Trp–Pro). Structural analysis reveals that A‐type HDGF mutant (P24A) apparently impact its ability in DNA binding and protein–protein interaction (Hung *et al*., 2015). Therefore, we mutated the Pro residue in P‐type HDGF (WT) to Ala (P24A mutant) and evaluated the effect on gene expression. We stably expressed P‐type HDGF (WT) or A‐type HDGF mutant (P24A mutant) in HDGF knockdown HepG2 cells. The A‐type HDGF mutant was less abundant in the nucleus than the P‐type HDGF (WT) was (Fig. 4A). This led us to hypothesize that the type of PWWP domain may affect the role of HDGF in regulation of gene expression. RNA‐Seq was performed in HepG2 cells stably expressing P‐type HDGF (WT) or A‐type HDGF mutant (P24A) to analyze regulation of gene expression by HDGF in relation to PWWP domain type. To gain insights into the molecular pathways coregulated by HDGF and PWWP domain type, we applied GSEA to identify potential pathways commonly perturbed by loss of HDGF and mutation of PWWP domain from A‐ to P‐type. Many overlapping pathways were disrupted by HDGF knockdown and A‐type HDGF mutant (P24A; Fig. 4B). As transcription activators, about 54% of the pathways activated by HDGF were downregulated by A‐type HDGF mutant (59%), suggesting that PWWP domain status is critical for HDGF regulation of gene transcription. GESA strongly indicated that fatty acid and steroid biosynthetic pathways were regulated by PWWP domain status (Fig. 4C–F). HDGF knockdown in HepG2 cells resulted in decreased enrichment of SERBP targets or genes activated by SREBP (Fig. 4G,H). SREBP target profile characteristics of loss of HDGF and mutating PWWP domain from A‐ to P‐type were similar, suggesting that HDGF regulation of SREBP‐mediated gene transcription is related to its PWWP domain. HDGF knockdown in HepG2 cells resulted in SREBP‐mediated transcriptional signatures similar to those in A‐type HDGF mutant expressed in HepG2 cells (Fig. 4I,J). qPCR confirmed that mutating PWWP domain from A‐ to P‐type resulted in suppression of expression of SREBP‐1 targets in HepG2 cells (Fig. 4K). Consistently, A‐type HDGF mutant (P24A) led to a decrease in SREBP‐1 targets in SMCC‐7721 cells (Fig. 4K), indicating a critical function of PWWP domain of HDGF in regulating SREBP1‐mediated lipogenic gene transcription.

### 3.5 HDGF stimulates SREBP1‐dependent gene expression by blocking recruitment of C‐terminal binding protein (CTBP)1 transcription repressor

**Figure 5:**
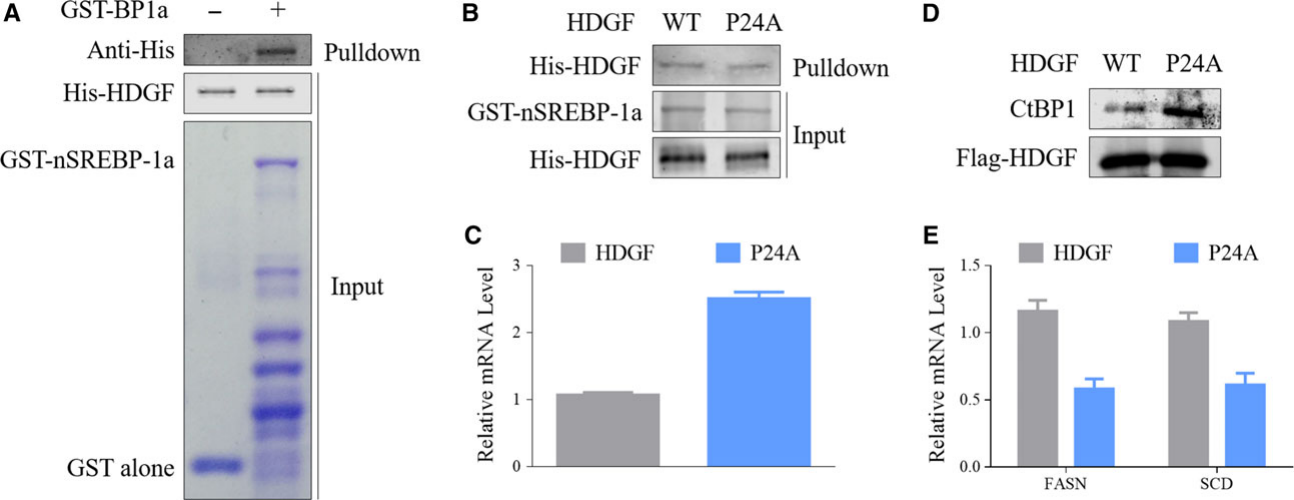
HDGF regulates recruitment of CTBP1 in gene transcription. (A) Recombinant GST–nSREBP‐1a or GST alone was incubated with purified His‐HDGF protein for 3 h. Bound proteins were eluted and analyzed with SDS/PAGE and Coomassie blue staining. Anti‐His antibody was used to detect HDGF in elutes. (B) Recombinant GST‐nSREBP‐1a was incubated with purified WT or P24A mutant (P24A) of His‐HDGF proteins for 3 h. Anti‐His antibody was used to detect HDGF in elutes. (C) ChIP assay was performed using antibodies against Flag in HepG2 cells stably expressing Flag‐HDGF and Flag‐P24A. SCD promoter sequences were amplified with qPCR. Error bars represent means ± SD (*n* = 3). (D) HepG2 cells stably expressing HDGF and P24A were cultured for 48 h. Cell lysates were prepared in IP buffer and immunoprecipitated with anti‐Flag antibody. The presence of CtBP1 was analyzed with immunoblotting using anti‐CtBP1 antibody. (E) ChIP assay was performed using antibodies against SREBP‐1 in HepG2 cells stably expressing Flag‐HDGF and Flag‐P24A. FASN and SCD promoter sequences were amplified with qPCR. Error bars represent means ± SD (*n* = 3).

To regulate SREBP‐mediated transcription, several transcriptional activators and repressors interact with SREBP proteins (Yang *et al*., 2006). Using GST pull‐down assay, we observed that HDGF directly interacted with SREBP‐1a (Fig. 5A), suggesting that HDGF directly participated in SREBP‐mediated transcription. However, A‐ or P‐type PWWP domain had no impact on the association between HDGF and SREBP‐1a (Fig. 5B). Thus, the binding of HDGF to SREBP may regulate gene transcription by influencing the recruitment of other transcriptional cofactors. HDGF1 reduces SYMD1 expression by recruiting the transcriptional repressor CTBP1 to the promoter (Yang and Everett, 2007). The promoter region of *SCD1* also contains an HDGF binding sequence similar to that of *SYMD1*. Therefore, we hypothesized that transcriptional inhibition of P‐type HDGF may be associated with recruitment of CTBP1. We analyzed the effect of the PWWP domain on HDGF enrichment in the promoter region of the SREBP‐1 target gene. A‐type HDGF mutant displayed stronger binding to the promoter region of SCD1 gene in contrast with WT P‐type HDGF (Fig. 5C). When the PWWP domain was A‐type, the interaction between HDGF and CTBP1 was significantly enhanced (Fig. 5D). In addition, enrichment of SREBP‐1 on the promoter region of the target gene was suppressed due to mutation of A‐type HDGF (Fig. 5E), suggesting that SREBP‐mediated transcription was blunted in that case. These data suggest that P‐type HDGF inhibits SREBP‐mediated transcription through binding and recruitment of transcriptional repressor CTBP1 to the promoter region of the target gene. The PWWP domain of HDGF is essential for promoting SREBP‐1‐mediated gene transcription.

### 3.6 HDGF P‐type inhibits lipid biosynthesis and cell proliferation

**Figure 6:**
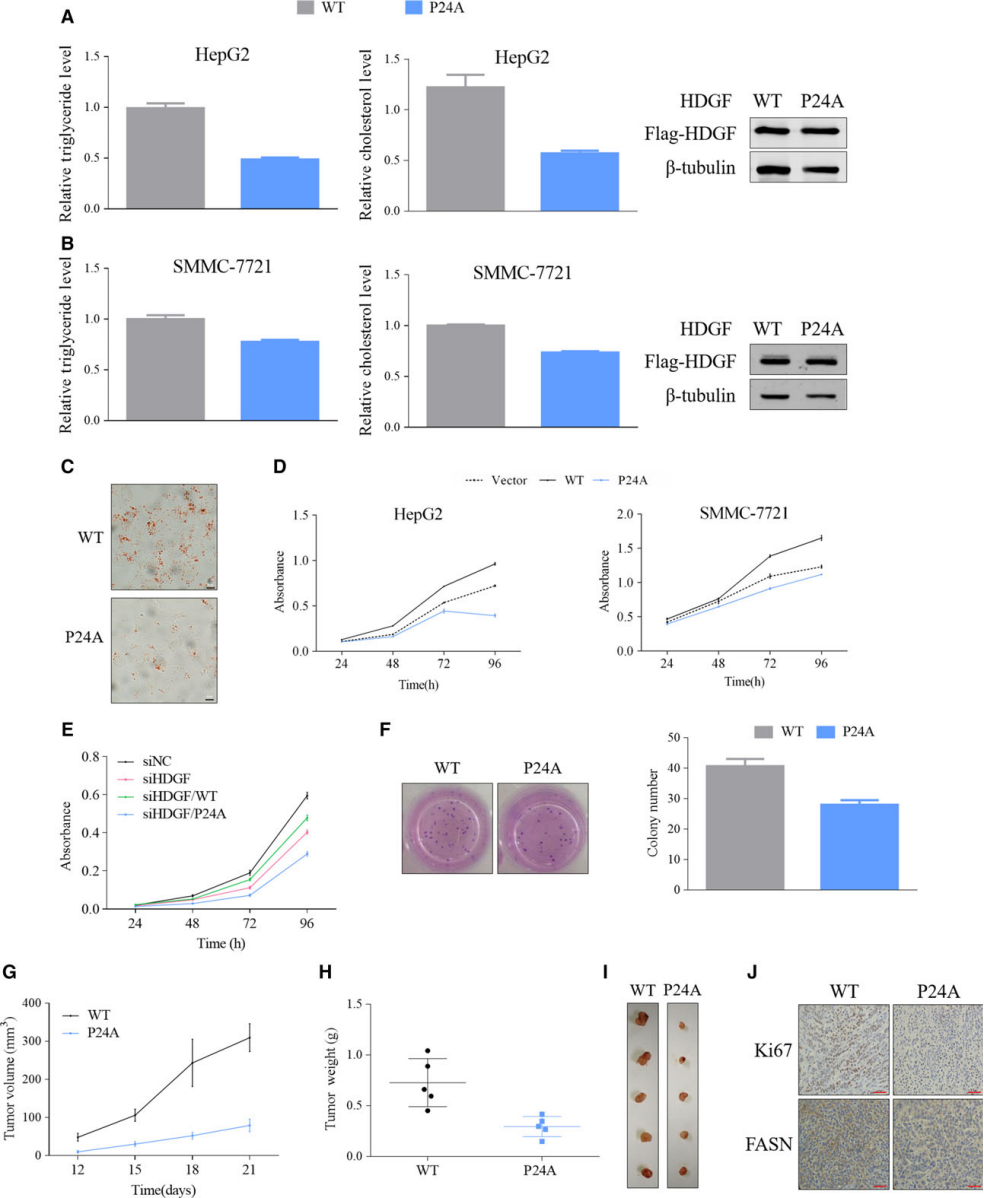
HDGF promotes lipid biosynthesis and cell proliferation of HCC cells. (A) Triglyceride or cholesterol levels were analyzed in HepG2 cells stably expressing HDGF and P24A. Lipid levels were normalized by total protein levels and the levels in control cells. (B) Cellular triglyceride or cholesterol levels were analyzed in SMC‐7721 cells stably expressing HDGF and P24A. Lipid levels were normalized by total protein levels and the levels in control cells. (C) HepG2 cells stably expressing HDGF and P24A were used for Oil Red O staining (scale bar, 20 μm). (D) Proliferation was analyzed in HepG2 or SMMC‐7721 cells stably expressing HDGF and P24A (vector as a control). Cell viability was analyzed every 24 h. (E) HepG2 cells were cotransfected with siRNA‐HDGF (siRNA‐NC as a control) and HA‐HDGF (WT: WT or P24A: P24A mutant). Cell viability was analyzed every 24 h. (F) HepG2 cells stably expressing HDGF and P24A were used for colony formation assay. Cells were cultured for 14 days. Crystal violet stained cells are presented on the right, and the colony numbers are on the left. (G) SMMC‐7721 cells stably expressing HDGF and P24A were injected subcutaneously into the dorsal flank of nude mice. The tumor volume was recorded at the indicated time. (H) When the mice were killed, the tumor weight was recorded. (I) The pictures of tumor were shown. (J) The tumor tissues were stained by Ki‐67 and antibodies against FASN (scale bar, 50 μm).

Since the levels of lipogenic enzymes were associated with the PWWP domain of HDGF, we analyzed whether changes in the PWWP domain type affected lipid metabolism in cancer cells. Compared with A‐type HDGF, P‐type HDGF suppressed biosynthesis of triglyceride and cholesterol (Fig. 6A,B). Oil Red O staining showed a significant reduction in the amount of lipid droplets in cells expressing P‐type HDGF (Fig. 6C). Many studies have shown that lipid and cholesterol biosynthesis is essential for cancer cell proliferation(Fritz and Fajas, 2010; Hirsch *et al*., 2010; Jiang *et al*., 2006). We hypothesized that changes in the P‐type to A‐type PWWP domain in HDGF would inhibit tumorigenesis. HDGF is known to play important roles in HCC cell proliferation(Bao *et al*., 2014b). Consistently, A‐type HDGF (i.e., WT) promoted proliferation of HCC cells, whereas P‐type HDGF mutant suppressed cell proliferation (Fig. 6D). In contrast, HDGF knockdown suppressed growth of HCC cells (Fig. 6E). Expression of A‐type HDGF (WT) reversed growth inhibition induced by HDGF knockdown, whereas P‐type HDGF mutant failed to do so (Fig. 6E). Consistently, the transition from A‐ to P‐type inhibited the ability of HDGF to promote HCC cell colony formation (Fig. 6F). We injected HCC cells stably expressing WT or P24 mutant into nude mice to analyze their effects *in vivo*. The tumor with expression of P‐type HDGF grew more slowly than the tumor with A‐type HDGF (Fig. 6G). The tumor was smaller in the P‐type than A‐type group. The tumor weight displayed a similar trend (Fig. 6H,I). The growth inhibition induced by P‐type HDGF was confirmed by Ki67 staining (Fig. 6J). The level of *Fasn*, one of the SREBP‐1 target genes, was decreased in tumors harboring P‐type HDGF, which was consistent with previous findings that PWWP status regulated SREBP‐1‐mediated gene transcription (Fig. 6J). Therefore, these data indicate that the status of the PWWP domain is important for HDGF in regulating growth of HCC cells.

### 3.7 Coexpression of SREBP‐1 and nuclear HDGF predicts poor prognosis of HCC

**Figure 7:**
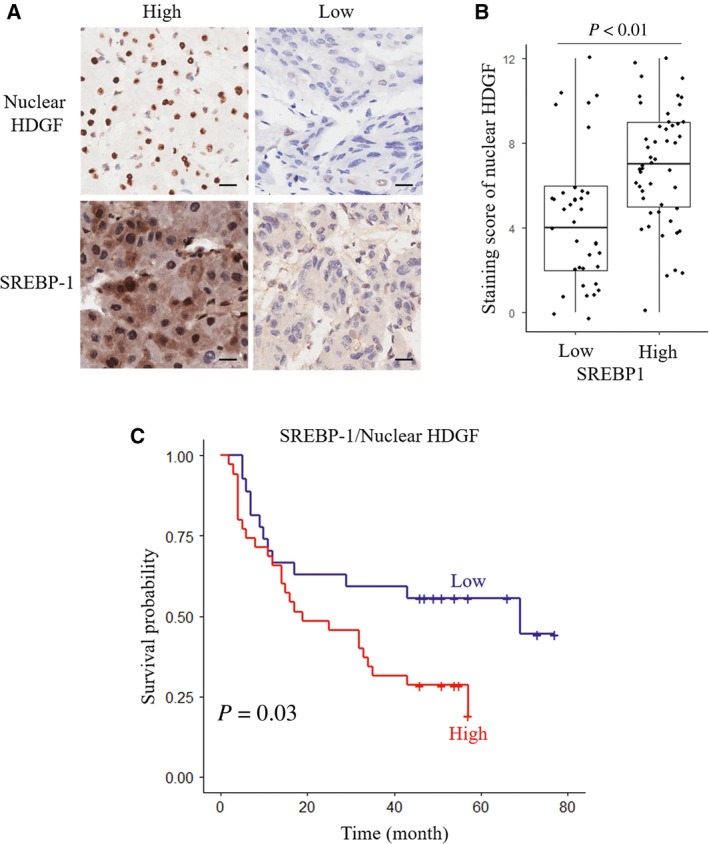
Coexpression SREBP‐1 and nHDGF predicts poor prognosis of HCC. (A) Representative images of a pair of HCC tissues (left) and adjacent normal tissues (right) stained with HDGF or SREBP‐1 antibody (scale bar: 20 μm). (B) Statistical analysis of levels of nHDGF with high and low expression of SREBP‐1 in HCC tissues. Staining score: 4.378 ± 3.156 and 6.362 ± 2.725 for SREBP1 low and high, respectively. (C) Kaplan–Meier analysis of OS rates for HCC patients with high or low expression of both SREBP1 and nHDGF.

We analyzed the relationship between SREBP‐1 and nHDGF in HCC tissues. The SREBP1 and nHDGF levels were positively correlated (*P* < 0.01). The increased expression of SREBP‐1 was accompanied by high nHDGF expression (Fig. 7A,B). Based on their levels, we divided the HCC patients into two groups that high group indicates the expression of HDGF and SREBP‐1 are both high‐level and low‐level group indicating the expression of HDGF and SREBP‐1 are both negative. HCC patients with coexpression of nHDGF/SREBP‐1 displayed poorer survival than patients without coexpression of nHDGF/SREBP‐1 (Fig. 7C). Therefore, these data indicate that coexpression of SREBP‐1 and nHDGF is a prognostic marker in patients with HCC.

## 4 Discussion

Hepatocellular carcinoma is one of the most common malignancies worldwide. The median OS of HCC is 4 months, and the 5‐year OS is 3%. Although many molecules have been proposed as potential targets for anticancer therapy, so far only sorafenib has been shown to improve median survival in advanced HCC patients (Cheng *et al*., 2009; Llovet *et al*., 2008). In our study, we found that HDGF expression was higher in cancer tissues than in adjacent noncancerous tissues. HDGF knockdown inhibited HCC cell growth. Our findings indicate that HDGF may be a candidate gene for the development of diagnostic and therapeutic strategies for HCC.

It is reported that HDGF is an important regulator of many cancer cell activities during transformation, apoptosis, angiogenesis, and metastasis (Bao *et al*., 2014b). There are mainly two different signaling pathways for growth factors. Induction of a typical kinase pathway at the plasma membrane leads to an intracellular phosphorylation cascade. It has recently been demonstrated that growth factors act as transcriptional cofactors in the nucleus. Although HDGF has been identified as a growth factor, its receptor on the cell membrane remains unclear. Nucleolin has been identified and validated as a HDGF‐interacting membrane protein. As a result, HDGF activates the downstream PI3K/AKT signaling pathway by binding to nucleolin (Chen *et al*., 2015a). The consensus sequence of the bidirectional nuclear localization signal is found in both the PWWP domain and other regions of the HDGF sequence. Nuclear localization of HDGF is essential for the mitogenic activity of HDGF in cells(Kishima *et al*., 2002). Our results suggest that the location of HDGF is strongly related to the poor prognosis of patients with HCC. After translocation into the nucleus, HDGF interacts with SREBP‐1 to initiate expression of genes involved in lipid metabolism. Thus, nucleus‐localized HDGF plays important roles in initiating lipid biosynthesis. In addition to the lipid metabolism genes, RNA‐Seq revealed that expression of many genes, such as those involved in cell division and spindle assembly, is also regulated by HDGF. Therefore, nHDGF may interact with other transcription factors, thus affecting the expression of many cancer‐related genes.

Hepatoma‐derived growth factor consists of a highly conserved N‐terminal PWWP module and a disordered C‐terminal 140‐residue domain and is homologous with high‐mobility group (HMG) proteins in the primary sequence that participate in transcriptional regulation (Bao *et al*., 2014b). The PWWP domain often involves chromatin‐associated biological processes (Qin and Min, 2014). The PWWP domain was first characterized from the WHSC1 (Wolf–Hirschhorn syndrome candidate 1) gene and was previously known as the HATH domain (Bao *et al*., 2014b). Proteins that contain the PWWP domain are involved in transcriptional regulation, DNA methylation, histone modification, and DNA repair by interacting with histone, double‐stranded DNA, and negatively charged molecules such as heparin (Chen *et al*., 2018; Hung *et al*., 2015; Rona *et al*., 2016). The type of PWWP domain modulates its binding and protein–protein interaction (Hung *et al*., 2015). We found that mutation from P‐ to A‐type PWWP domain affected gene expression, especially genes involved in lipid metabolism. A‐type HDGF was less abundant in the nucleus than P‐type HDGF. Thus, the PWWP domain plays a critical role in HDGF regulation of gene transcription. The PWWP domain of HDGF consists of a five‐stranded antiparallel β‐barrel followed by two α‐helices and is involved in protein–protein, protein–RNA, and protein–DNA interactions (Bao *et al*., 2014a). Changes in the protein structure induced by the first mutation of the PWWP motif may affect nuclear translocation and DNA–protein binding of HDGF.

Mutations in the PWWP domain are associated with a variety of human diseases. Lens epithelial cell‐derived growth factor plays an important role in tethering HIV‐1 cDNA to human chromatin through the PWWP domain (Blokken *et al*., 2017). Disruption of the PWWP domain of the WHSC1 protein results in lymphoid multiple myeloma(Stec *et al*., 2000). The S144I mutation in the PWWP domain of mismatch repair protein 6 leads to hereditary nonpolyposis colorectal cancer(Laguri *et al*., 2008). A missense mutation in the PWWP domain of mammalian DNA methyltransferase 3B is closely related to immunodeficiency, centromeric heterochromatin instability, facial anomalies syndrome (Ge *et al*., 2004). Our data indicate that the first amino acid mutation in the PWWP domain inhibits HCC cell proliferation and tumorigenesis, confirming the important role of the PWWP domain in biological processes.

**Figure 8:**
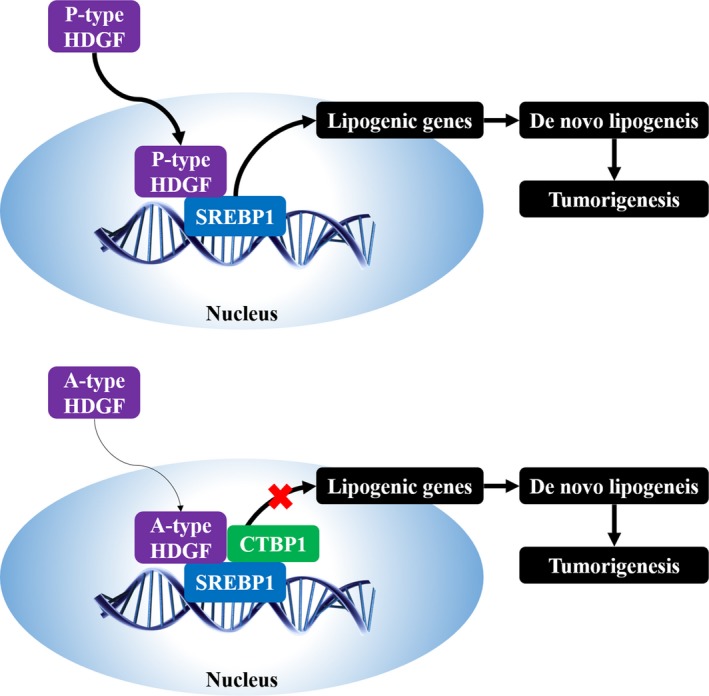
Working model describing the role of HDGF in the regulation of lipogenesis and oncogenesis.

Cellular lipids, especially cholesterol and fatty acids, serve both as basic structural components of cell membranes and as metabolic intermediates and signaling factors in networks that coordinate most biological processes. In addition to the Warburg effect, increased *de novo* lipogenesis is thought to be another major metabolic change in cancer cells (Fritz and Fajas, 2010). Metabolic reprogramming to activate *de novo* lipogenesis is essential for cancer development. Highly activated *de novo* lipogenesis is a key feature of HCC as well as other types of malignant tumor (Hirsch *et al*., 2010). HCC patients have higher levels of plasma free fatty acids than controls have, including saturated and monounsaturated fatty acids (Jiang *et al*., 2006). Targeting lipid metabolism and its related regulators have emerged as a promising antitumor strategy. We have previously found that SREBP1‐mediated *de novo* lipogenesis promotes cancer cell proliferation by providing sufficient lipids (Liu *et al*., 2016; Zhu *et al*., 2016). Transcription factors require cofactors to help fully activate gene transcription. At the same time, these transcriptional cofactors control specific gene transcription. A mediator complex including ARC105 and CDK8 has been shown to be essential for SREBP control of cholesterol and fatty acid homeostasis (Yang *et al*., 2006; Zhao *et al*., 2012). In this study, we found that HDGF promoted SREBP‐1‐mediated gene transcription via blocking recruitment of CTBP1 transcription repressor in HCC cells (Fig. 8). As HDGF promotes cell division and proliferation, there is a sudden increase in the demand for lipids. Our study may provide specific mechanisms by which HDGF coordinates lipid metabolism and cell mitosis. Accumulating evidence indicates that SREBP is not only involved in lipid metabolism but also other biological processes (Shimano and Sato, 2017). It is possible that various functions of HDGF are involved in SREBP‐mediated transcription.

## 5 Conclusion

This study describes the mechanisms underlying HDGF promotion of tumorigenesis and lipid biosynthesis in HCC cells. HDGF promotes *de novo* lipogenesis through activating SREBP‐1‐mediated lipogenic gene transcription. We demonstrated that high HDGF and SREBP‐1 expressions were significantly associated with poor prognosis of patients with HCC. Therefore, targeting the actions of the HDGF/SREBP‐1 axis may represent a promising strategy for the development of effective agents for HCC treatment.

## Author contributions

XM, GH, JL, and XZ conceived and designed the study. XM, LZ, KW, JW, QL, and XZ performed the experiments. XM and XZ drafted the manuscript. GH and JL edited the manuscript.
